# Effects of Antibiotics and Anti-Inflammatory Drugs on Enamel Development: A Systematic Review with Quantitative Synthesis

**DOI:** 10.1016/j.identj.2026.109478

**Published:** 2026-03-12

**Authors:** Nadia Zamri, Hien Ngo, Elizabeth Court, Lakshman Samaranayake, Kausar Sadia Fakhruddin, Bennett Tochukwu Amaechi, Saniya Khani

**Affiliations:** a Pharmacy, Charleville Hospital, South West Hospital and Health Service, Queensland, Australia; b UWA Dental School, The University of Western Australia, Nedlands, Perth WA, Australia; c Department of Clinical Pharmacology, Royal Brisbane and Women’s Hospital, Queensland, Australia; d Dr DY Patil Dental College and Hospital, Dr DY Patil Vidyapeeth, Pimpri, Pune, India; e Faculty of Dentistry, University of Hong Kong, Hong Kong; f Cariology Unit, Department of Community Dentistry, University of Texas Health Science Centre at San Antonio, San Antonio, TX, USA; g Los Medanos College, Brentwood, California, USA

**Keywords:** Enamel matrix, Antibiotic, NSAIDs, Biomineralisation, Systematic review

## Abstract

**Objective:**

To investigate the effects of commonly prescribed antibiotics and nonsteroidal anti-inflammatory drugs (NSAIDs) during early life on enamel development through a systematic review with quantitative synthesis.

**Methods:**

PubMed, Web of Science, Embase, CINAHL, and Scopus were searched for English-language studies published between 1995 and 2024. *In vitro* and *in vivo* experimental studies evaluating antibiotics and/or NSAIDs during tooth development were included. Quantitative meta-analysis was restricted to *in vivo* animal studies reporting comparable outcomes for enamel thickness or mineral content, while *in vitro* studies contributed to qualitative mechanistic synthesis. Studies focusing solely on dentine, using questionnaires, lacking extractable data, or classified as grey literature or reviews were excluded.

**Results:**

Experimental exposure to antibiotics and NSAIDs was associated with alterations in ameloblast morphology and function, including changes in tight junction proteins, MMP-20, KLK4, COX-2, and Runx2 expression. Enamel matrix abnormalities such as vacuolar changes and cyst-like lesions were frequently reported. Quantitative synthesis indicated a moderate-to-large pooled effect estimate for reduced enamel thickness associated with amoxicillin exposure (Cohen’s d = 0.79, 95% CI: 0.40-1.36), with a trend toward larger effect sizes at higher experimental doses; these estimates were interpreted cautiously due to substantial heterogeneity. NSAIDs were primarily associated with qualitative disturbances in enamel mineralization. Pooled effect estimates for calcium (Cohen’s d = 0.52, 95% CI: −0.21 to 1.46) and phosphorus (Cohen’s d = 0.46, 95% CI: −0.31 to 1.75) were inconsistent and imprecise.

**Conclusions:**

Certain antibiotics and NSAIDs may disrupt ameloblast integrity and enamel biomineralization in experimental animal models. These findings are hypothesis-generating, and further well-designed human observational and mechanistic studies are required before clinical translation. *Clinical Relevance:* This systematic review synthesizes experimental evidence on the biological vulnerability of developing enamel to pharmacological exposure, highlighting potential mechanisms underlying enamel thinning and hypomineralization while underscoring the need for cautious interpretation when extrapolating animal data to human clinical contexts.

PROSPERO Registration No.: CRD42025606606.

## Introduction

Dental development occurs both pre- and postnatally and continues, albeit to a lesser degree, throughout life with the deposition of secondary and tertiary dentin.^1^^,^^2^ The development of deciduous teeth begins between the sixth and seventh weeks of intrauterine life, while permanent teeth formation starts at the 14th week of gestation and continues up to 5 years after birth.^3^ Tooth formation involves complex interactions between the oral epithelium and dental mesenchyme, progressing through the bud, cap, and bell stages. Each stage of tooth development is characterized by specific morphological changes that shape the tooth crown.^4^^,^^5^

During the bud stage, epithelial and mesenchymal tissues interact to determine tooth morphology.^6^ The cap stage involves the differentiation into inner and outer enamel epithelium layers, while the bell stage sees the formation of the enamel organ, with capillary plexuses supplying nutrients to enamel organ cells.^6^ This stage includes morphodifferentiation, which shapes the crown, and histodifferentiation, where inner enamel epithelial cells differentiate into ameloblasts and odontoblasts. Odontoblasts produce the dentinal matrix, whereas ameloblasts are responsible for the tooth enamel matrix formation.^7^

Enamel, the hardest mineral structure in the human body,^8^ develops through a process called amelogenesis. This is a complex biological process divided into pre-secretory, secretory, transition, and maturation phases. In the pre-secretory phase, inner enamel epithelial cells differentiate into ameloblasts.^8^ These specialized cells deposit an organic matrix composed predominantly of amelogenins and other non-amelogenin proteins during the secretory phase, forming a scaffold that later mineralizes into hydroxyapatite crystals. The Tomes’ process, a structural extension of ameloblasts, is crucial in directing the secretion and structure of the enamel matrix.^8^

Cyclooxygenase-2 (COX2) plays a vital role in enamel maturation by mediating inflammation and regulating blood flow and nutrient delivery to ameloblasts.^9^ Carbonic anhydrase helps buffer the increased acidity from crystal growth despite an overall decrease in pH.^8^ Key enzymes such as Matrix Metalloproteinase-20 (MMP-20) and Kallikrein-4 (KLK4) are essential in this process. MMP-20 is active during the early secretion and maturation phases and is responsible for degrading matrix proteins to facilitate hydroxyapatite crystal growth and increase enamel hardness.^10^^,^^11^ KLK4 functions in the late maturation stage, degrading residual enamel proteins to enhance mineral content and crystal thickening.^11^ Additionally, junctional proteins such as Claudins (CLDNs) and Occludin (OCLN) in ameloblast tight junctions regulate paracellular permeability, maintaining the microenvironment for enamel maturation.^12^ Genetic factors, such as Runx2, are crucial for regulating enamel protein accumulation and enzyme synthesis; their absence can delay mineralization.^8^

The prolonged maturation phase, which spans several years in humans, is facilitated by the ameloblasts’ machinery and the fenestration of capillaries near the enamel surface, allowing ample time for ion diffusion.^8^ Enamel development can be perturbed by various environmental influences and genetic alterations. Amelogenesis is a highly regulated process and can be negatively influenced by pathological or medical conditions such as fever, infection, trauma, changes in oxygen saturation, antibiotics, and many other factors.^13^ The complexity of enamel formation and its susceptibility to numerous environmental factors underscore the importance of understanding these interactions for dental health.

In general, enamel defects are common in the general population,^14^^,^^15^ and could be as high as 50% of the populace, depending on the fluoride content of their drinking water. These defects include enamel hypoplasia, a deficiency in the quantity of enamel, versus qualitative color changes leading to hypomineralization.^15^^,^^16^ Not all teeth, or even all surfaces of teeth, are equally affected by enamel defects.^14^

Several studies^17^, ^18^, ^19^ have indicated that some commonly prescribed anti-inflammatory drugs (such as acetaminophen, ibuprofen) and antibiotics for neonates and children under 5 years old can interfere with enamel biogenesis. Understanding the intrinsic biological processes and the extrinsic influences during amelogenesis is crucial for developing effective remedies and preventive measures for enamel defects. Due to limited data on this topic, this systematic review aims to evaluate the impact of antibiotics and anti-inflammatory medications on developing dentition and enamel demineralization using existing animal model studies in the literature.

## Methodology

Two investigators (NZ and KSF) searched English-language manuscripts published between January 1995 and October 2024 in PubMed, Web of Science, Embase, CINAHL, and Scopus to assess the impact of commonly prescribed medications on children’s developing enamel. The search timeframe (1995-2024) was selected to capture the period during which modern antibiotics, NSAIDs, fluoride exposure paradigms, and contemporary enamel biomineralization assessment techniques were established, ensuring methodological relevance and comparability across included studies.

Employing the PICO framework, a specific review question was formulated: Does the medication (NSAIDs/Acetaminophen/Amoxicillin/Amoxicillin+Clavulanate/Ampicillin/Gentamicin/Macrolides-Erythromycin/Tetracycline/Azithromycin (I) compared to no medications (C) impact on ameloblast morphology, enamel matrix, and enamel biomineralization (O) on the developing enamel (P)? The search keywords were organized according to the PICO model, attached as a supplementary file.

### Inclusion criteria

• *In vitro* experimental studies.
• *In vivo* experimental studies.
• Any class of antibiotic and/or anti-inflammatory drugs, medications alone or with fluoride.
• Country or date enforced no limitations.

### Exclusion criteria

• Studies including the effect of medications on the developing dentine only
• Clinical studies using questionnaire data to assess the impact of medications on children’s teeth
• Review articles
• Articles that do not allow data extraction required to meet the set objectives
• Studies with incomplete outcome details
• Grey literature and unpublished information were neither considered nor used.

**Fig. 1:**
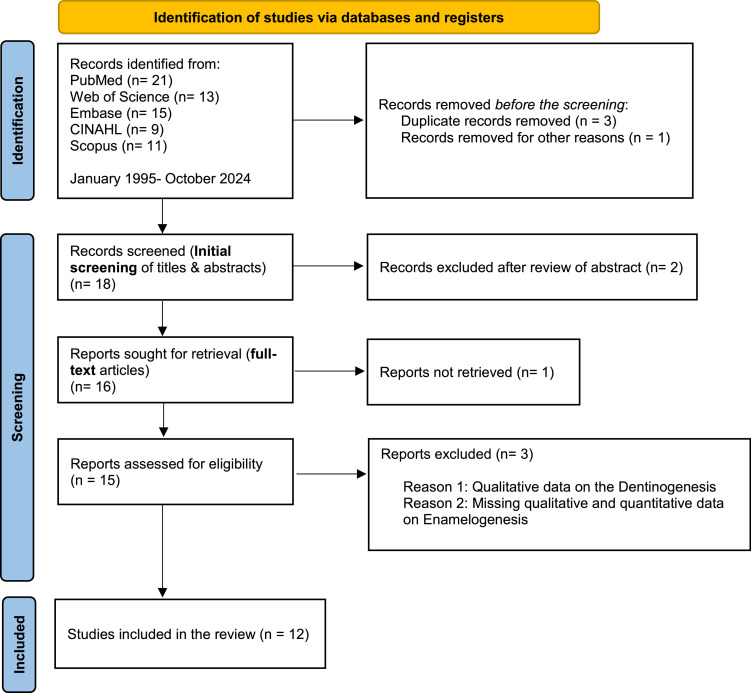
PRISMA flow chart of the literature search and study selection.

The study’s primary outcome was the effect of medications on enamel biogenesis. We followed the Preferred Reporting Items for Systematic Reviews and Meta-Analyses guidelines (PRISMA)^20^^,^^21^ to ensure a systematic, transparent, and comprehensive process (Figure 1). The identified research articles were compiled using the bibliographic software EndNote 12 (Clarivate Analytics, USA).

The data search and analysis were carried out in three stages. The titles and abstracts of all relevant studies that met the predetermined inclusion criteria were systematically screened during stage one. The full-text review was included when there was a complete data set available to support accurate decision-making. In stage two, for the full-text review, the investigators used spreadsheets to ensure that the eligibility criteria were met and the outcomes that fulfilled the study objectives were reported and retrievable.

References were checked using a backward search of the included studies. A manual search was conducted on the references listed in the included studies to identify additional studies that could be considered. The reviewers systematically extracted and analyzed the data in the third and final stages, Table 1. To address methodological heterogeneity across included studies, outcomes were pre-specified and categorized into four analytical domains: (1) ameloblast morphology and histopathological alterations, (2) enamel matrix protein and enzyme changes (including MMP-20, KLK4, COX-2, and Runx2), (3) enamel mineralization outcomes (calcium and phosphorus content), and (4) enamel thickness. This structured categorisation guided data extraction, synthesis, and interpretation, enabling clearer comparison across diverse experimental designs and exposure models.

**Table 2:** Characteristics of the included studies and the results.

Study Year Country	Study design Employed methodology	Test medication	Stage of enamel development	Biological mechanism/s	Analysis technique/s	Primary outcome
**Anti-Inflammatory medications**						
**Gonçalves J de Lima et al 2022** Brazil	An experimental study Sixty male mice were divided into three groups. Gr 1: 20 (Celecoxib) Gr 2: 20 (Indomethacin) Gr 3: 20 (control)	NSAIDs: **Celecoxib** **Indomethacin** NM	Effects of NSAIDs during the secretion and maturation phases of amelogenesis	Impact on the enzymatic activity and expression of MMP-20 Changes in COX-2 expression Inhibition of the synthesis of transcription factor Runx2	Micro-computed tomography Scanning electron microscopy Energy dispersive X-ray analysis Knoop microhardness testing Immunohistochemistry *In situ* zymography Indirect immunofluorescence	NSAID drugs reduce enamel Ca and P content, microhardness, and mineral density, thereby affecting enamel biomineralization. NSAIDs impair the synthesis and nuclear translocation of proteins and enzymes important for enamel formation.
Serna Muñoz et al **2018** Spain	Forty-two Swiss mice were divided into two main and seven subgroups, including the control group. Gr 1= Antibiotics Gr 2= Anti-inflammatory Subgroups: G2a =Acetaminophen G2b= Ibuprofen G2c = Celecoxib	NSAID: **ibuprofen group**, treated with 2.5 mg/d **The celecoxib group** was treated with 0.12 mg/d. The **Acetaminophen** group, treated with 5 mg/d	Effects of anti-inflammatories during the maturation phases of amelogenesis	Changes in COX-2 expression	Scanning electron microscopy Energy dispersive X-ray analysis Immunohistochemistry Image analysis quantification of immunoreactivity	Acetaminophen and celecoxib significantly decreased Ca and P compared with the control samples. Ca/P ratios showed no difference. Acetaminophen showed significantly lower levels of immunoreactive COX-2 at the maturation stage of the mouse incisors.
**Antibiotics**						
**Sabah and Al Ghaban** **2023** Iraq	16 pregnant adult Wistar rats were equally divided into two groups. Gr 1: *n* = 8 (Control) Gr 2: *n* = 8 (Experimental)	**Amoxicillin** Gr 1: Saline solution Gr 2: 250 mg/kg/d From the 13th gestational day until delivery, every day, by oral gavage. The newborns received the same treatment from the first day until 7 or 12 d after birth.	Effects during the secretory (day 7) and early maturation stages (day 12) of enamel formation	Amoxicillin interferes with the early phases of amelogenesis by altering the structure of ameloblasts and decreasing the enamel matrix.	Histomorphometric Histological analysis	The ameloblast and odontoblast layer thickness were significantly different between the groups. Vacuolization of the ameloblastic and odontoblastic layers was observed in the treated group in antenatal and post-natal durations.
**Schmalfuss et al 2022** Norway	Neonatal mice were randomized into two groups: Gr 1: *n* = 36 (Experimental) Gr 2: *n* = 35 (Control)	Gr 1: Study group **Ampicillin**, solution (200 mg/kg) 2.0 μl/g mouse body weight **Gentamicin**, solution (4 mg/ kg) 1.3 μl/g mouse body weight Antibiotics were injected intravenously for 4 d. Gr 2: Control (sterile saline)	Enamel secretory stage	NM	Macro photography Micro-CT imaging	A significantly lower vol% enamel in both maxillary (30.9% vs 32.7*%; P = .004*) and mandibular (32.5% vs 34.6%; *P = .015)* molars in the experimental than in the control groups, respectively).
**Feltrin de Souza, J. et al 2021** Brazil	Forty rats were randomly assigned to four groups: Gr 1: Amoxicillin group Gr 2: Fluoride group, Gr 3: Amoxicillin + fluoride group Gr 4: Control group	**Amoxicillin** (500 mg/kg/d), **Fluoridated water** (100 ppm −221 mg F/L)	NM	NM	Dental Fluorosis by Image Analysis (DFIA) software Microhardness tester and Knoop indenter Ion-selective electrode coupled to a potentiometer	The rats exposed to fluoride or fluoride + amoxicillin developed dental fluorosis. Exposure to amoxicillin alone did not lead to enamel defects.
**Gao, J. et al 2020** China	Thirty-six 3-d-old Kunming mice were randomly divided into three groups. Gr 1: (Control group) Gr 2: 50 mg/kg Gr 3: 100 mg/kg	**Amoxicillin** 50, or 100 mg/kg amoxicillin by intragastric administration for 19 d.	Effects during the secretory and maturation stages	Amoxicillin reduced the expression of tight junction proteins (CLDN1, CLDN4, and OCLN) in mature ameloblasts, influencing tight junctions in cells during enamel maturation, affecting the paracellular permeability and microenvironment for enamel mineralization. Amoxicillin also decreased KLK4 enzyme expression and induced cyst-like lesions.	Scanning electron microscopy Energy dispersive X-ray spectroscopy Immunohistochemical staining	Amoxicillin decreased the Ca/P ratio in the enamel of mandibular incisors and molars. Intercellular spaces among maturation ameloblasts were observed in the amoxicillin-treated groups.
**Kameli et al 2019** Iran	Twelve pregnant adult Wistar rats were equally divided into four different groups. Gr 1: *n* = 3 (negative control) Gr 2: *n* = 3 (positive control) Gr 3: *n* = 3 (Amoxicillin group) Gr 4: *n* = 3 (Amoxicillin group)	**Amoxicillin** **Tetracycline** Gr 1: Saline solution. Gr 2: Tetracycline (130 mg/kg). Gr 3: Amoxicillin 50 mg/kg (every 8 h) Gr 4: Amoxicillin 100 mg/kg (every 8 h) The treatments were administered daily from the 13th gestation day to the end of gestation.	NM	Dentin vacuolization of the odontoblastic layer was observed in the tetracycline- and amoxicillin-treated groups.	Histological analysis Histomorphometric examination using the morphometric analysis software	The mean ameloblastic and odontoblastic layer, enamel, and dentin thickness significantly differed in the experimental groups compared to the control.
**Munoz Clara et al 2018** Spain	Forty-two Swiss mice were divided into seven groups: Gr 1: Amoxicillin Gr 2: Amoxicillin/clavulanate Gr 3: Erythromycin Gr 4-6: Anti-inflammatory Gr 7: Control	**Amoxicillin** **Amoxicillin/clavulanate** **Erythromycin** Gr 1: Amoxicillin (5 mg/d) Gr 2: Amoxicillin/clavulanate (2.5/0.31 mg/d) Gr 3: Erythromycin (5 mg/d)	Effects during the enamel maturation stages	Significantly lower amounts of immunoreactive COX2 at the enamel organ maturation stage	Scanning electron microscopy–Energy dispersive X-ray analysis Immunohistochemistry Image analysis quantification of immunoreactivity	Antibiotic groups showed no significant differences in the content of Ca and P elements. Only acetaminophen and celecoxib significantly decreased Ca and P compared to the control. However, Ca/P ratios showed no difference. Groups treated with antibiotics and acetaminophen showed significantly lower amounts of COX2 at the enamel organ maturation stage.
**de Souza, J. F et al 2016** Brazil	Fifteen pregnant rats were randomly assigned to three groups Gr1: Physiological solution (sham group) Gr 2: Amoxicillin Gr 3: Amoxicillin After birth, each group received the same treatment until post-natal day 7 or 12	**Amoxicillin** Gr1: Physiological solution (sham group) Gr 2: Amoxicillin 100 mg/kg/ Gr 3: Amoxicillin 500 mg/kg/d	Enamel secretory (day 7) and early maturation stage (day 12)	Amoxicillin interferes with the initial stages of amelogenesis by causing structural changes (vacuoles) in the ameloblasts	Immunohistochemistry for amelogenin and MMP-20 detection	There is a reduced enamel matrix, which results in reduced enamel thickness. No significant differences were found in the number of amelogenin- and MMP-20-immunolabeled ameloblasts between the test and control groups.
**Mihalas E, et al 2016** Romania	Twenty-eight C57BL/6 male mice of similar age, randomly divided into a control and three treatment groups (*n* = 7). Gr 1: *n* = 7 Gr 2: *n* = 7 Gr 3: *n* = 7 Gr 4: Control	**Amoxicillin/Clavulanic Acid** Received subcutaneous injection of antibiotics for 60 d Gr 1: 50 mg/kg Gr 2: 100 mg/kg Gr 3: 150 mg/kg Gr 4: Control Received only solvent (0.1 ml sterile water once per day)	Enamel Maturation stage	Dose-dependent effects on ameloblast functions, especially in the maturation phase, causing quantitative and/or qualitative enamel defects	Scanning electron microscopy Energy dispersive X-ray spectroscopy Histological analysis	EDX analysis showed that treatment groups tended to show a lower content of Ca and P in enamel The group treated with 50 and 100 mg/kg showed quantitative defects in pits or fissures with variable incidence, size, and depth, usually with smooth, regular margins. The groups treated with 150 mg/kg showed signs of hypomineralization, not as individual lesions but associated with hypoplastic ones.
**Gottberg et al 2014** Venezuela	12 pregnant adult rats were used and distributed into five different groups: Gr 1: *n* = 2 (Control) Gr 2: *n* = 2 (Negative control) Gr 3: *n* = 3 (Tetracycline) Gr 4: *n* = 3 (Amoxicillin) Gr 5: *n* = 2 (Amoxicillin)	**Amoxicillin** **Tetracycline** Gr 1: Control Gr 2: Saline solution Gr 3: Tetracycline 130 mg/kg, Gr 4: Amoxicillin 50 mg/kg Gr 5: Amoxicillin 100 mg/kg From the 6th gestation day to the end of gestation, treatments were administered orally by mouth daily.	NM	A dose-dependent effect was observed during amelogenesis.	Histological analysis	Hypomineralization was observed in every tetracyclic and amoxicillin-treated group sample with a 100 mg/kg dose. Meanwhile, only 50% of the group administered 50 mg/kg amoxicillin showed histological disorder.
**Sahlberg C et al 2013** Finland	Mandibular molar tooth germs of E18 mouse embryos: First molar: *n* = 348 Control group: 83 Test group: 265 Second molar: *n* = 322 Control group: 76 Test group: 246 These are further subdivided into 12 groups.	**Amoxicillin** **Amoxicillin/clavulanate** **Sodium clavulanate** **Sodium Fluoride** Tooth germs were cultured for 10 d in the following medium Gr1: 0.07 mg/mL clavulanate Gr2: 0.5 mg/mL amoxicillin Gr3: 0.07 mg/mL clavulanate+0.5 mg/mL amoxicillin Gr4: 1 mg/mL amoxicillin Gr5: 2 mg/mL amoxicillin Gr6: 3.6 mg/mL amoxicillin Gr7: 10 mM NaF and 1 mg/mL amoxicillin Gr8: 12 mM NaF and 1 mg/mL amoxicillin Gr9: 12 mM NaF + 2 mg/mL amoxicillin Gr10: 12 mM NaF + 3.6 mg/mL amoxicillin Gr11: 15 mM NaF + 2 mg/mL amoxicillin Gr12: 15 mM NaF + 3.6 mg/mL amoxicillin	From the secretory stage to the early maturation stages of amelogenesis	The effects were dose-dependent and potentiated each other on ameloblast functions, from the secretory to the early maturation phase. Both amoxicillin and NaF decrease MMP20 expression.	Morphological changes from the tooth photographs. Histological tissue sections using the light microscope	For the first molar, NaF and/or amoxicillin dose dependently retard the transition of ameloblasts from the secretory stage to the maturation stage. Exposure to 3.6 mg/ mL and frequently to 2 mg/mL of amoxicillin shows enamel porosity. For the second molar, there was retarded enamel formation after exposure to 1 or 2 mg/mL of amoxicillin. Teeth exposed to 15 mM NaF or 3.6 mg/mL of amoxicillin alone or in combination show multiple vacuoles covering the enamel surface between the poorly organized ameloblasts.
**Abe et al 2003** Japan	Twenty animals, each of a sex-specific pathogen-free Jcl: Wistar rats, approximately 5 wk (weight 168-180 g) Gr 1: *n* = 10 Gr 2: *n* = 10	**Macrolides** A novel macrolide antibiotic (Code No. ML-100) Gr 1: Novel macrolide – once daily at a dose of 5000 mg/kg/d for 5 wk Gr 2: Control – Maintained without any treatment for 10 wk.	Effects on the enamels’ transitional, maturation, and pigmentation stages.	In the rats administered with ML-100, an increase in the number of karyopycnosis of ameloblasts was observed at the transitional stage. This karyopycnotic change may be caused by the involvement of inhibition of protein synthesis in the transformation of the ameloblasts.	Histopathology Contact microradiography (CMR)	The primary lesion induced by a novel macrolide antibiotic is the increased karyopycnosis of ameloblast at the transitional stage, followed by a later stage.

Ca, Calcium; COX, cyclooxygenase (COX) enzyme; NM, Not Mentioned; NSAIDs, Nonsteroidal anti-inflammatory drugs.

The reviewed manuscripts were assessed using SYRCLE’s Risk of Bias (RoB) tool,^22^ to evaluate the study’s methodological quality (Table 2). This tool encompasses ten items addressing the evaluation of the method of randomization, blinding of outcome assessment, allocation concealment, selective reporting, and other potential sources of bias. Adapted from the Cochrane RoB tool, this tool has been tailored to account for specific biases pertinent to animal intervention studies. In the third stage, the RoB assessment was conducted by two reviewers (KSF and NZ), and the discrepancies were discussed by two other reviewers (HCN and MD) until a mutual consensus was reached and documented as low, unclear, or high risk. Quantitative synthesis was restricted exclusively to *in vivo* animal studies with comparable outcome measures, whereas *in vitro* studies were used for qualitative synthesis.

**Table 1:** Risk of bias assessment.

Study	Selection bias			Performance bias		Detection bias		Attrition bias	Reporting bias	Other
	Sequence generation	Baseline characteristics	Allocation concealment	Random housing	Blinding	Random outcome assessment	Blinding	Incomplete outcome data	Selective outcome reporting	Other sources of bias
**Gonçalves et al****^23^**	Unclear	Yes	Yes	Yes	Yes	Unclear	Yes	Unclear	Unclear	No
Serna Muñoz, **et al**^24^	Unclear	Yes	Yes	Yes	Unclear	Unclear	Yes	Unclear	Unclear	No
**Sabah and Al Ghaban**^25^	Yes	Yes	Yes	Unclear	Unclear	Unclear	Yes	Unclear	Unclear	No
**Schmalfuss et al**^26^	Yes	Yes	Yes	Yes	Yes	Yes	Yes	Unclear	Unclear	No
**Feltrin de Souza et al**^27^	Unclear	Yes	Yes	Yes	Unclear	Unclear	Yes	Unclear	Unclear	No
**Gao et al**^28^	Yes	Yes	Yes	Yes	Yes	Unclear	Yes	Unclear	Unclear	No
**Kameli et al**^29^	Unclear	Yes	Unclear	Unclear	Unclear	Unclear	Yes	Unclear	Unclear	No
Serna Muñoz, **et al****^24^**	Unclear	Yes	Yes	Yes	Unclear	Unclear	Unclear	Unclear	Unclear	No
**de Souza et al**^30^	Yes	Yes	Yes	Yes	Unclear	Unclear	Yes	Unclear	Unclear	No
**Mihalas et al**^31^	Yes	Yes	Yes	Unclear	Unclear	Unclear	Yes	Unclear	Unclear	No
**Sahlberg et al**^32^	Yes	Yes	Yes	Yes	Yes	Yes	Yes	Unclear	Unclear	No
**Gottberg et al**^33^	Yes	Yes	Yes	Yes	Yes	Unclear	Yes	Unclear	Unclear	No
**Abe et al**^34^	Yes	Yes	Yes	Yes	Yes	Yes	Yes	Unclear	Unclear	No

We used SPSS macros (IBM Corp., Armonk, NY, USA) to calculate an aggregated mean ES, Cohen’s d, and 95% confidence intervals. The data from the included studies were divided into treatment and control groups. The treatment groups were sub-grouped based on differences in evaluated medications and dosages. Sample size, type of medications and dosages, mean and standard deviation (SD) of SEM-EDX (%wt) values for calcium (Ca) and phosphorus (P) content, enamel thickness, and statistical significance were recorded individually for each comparison.

When numerical data were not directly reported, and only graphical representations were available, data points were extracted using the WebPlotDigitizer software tool. Following extraction, mean values and SDs were calculated using Python software.

Two effects on the developing enamel were calculated: enamel thickness and Ca and P mineral contents. Only *in vivo* animal studies reporting quantitative outcomes for enamel thickness or mineral content were included in the meta-analysis; *in vitro* studies were synthesized narratively due to differences in experimental design and endpoints.

To estimate the magnitude of medication effects, effect sizes (ES) were computed as Cohen’s d, defined as the mean difference between the experimental and control groups divided by the pooled SD. A random-effects model was applied to account for variability across studies arising from differences in experimental conditions, including duration of exposure, animal species, medication type, dosing regimens, and co-exposure to fluoride. Using a random-effects model, aggregated effects were calculated using inverse-variance weighting to account for within-study precision and between-study variability. Pooled estimates were interpreted as exploratory indicators of relative magnitude and direction rather than confirmatory effect estimates. Effect sizes were interpreted using conventional thresholds (small = 0.2, moderate = 0.5, large = 0.8).

Statistical heterogeneity was assessed using the *I²* statistic, with values of 0% to 25% indicating low heterogeneity, 26% to 50% moderate heterogeneity, 51% to 75% substantial heterogeneity, and 76% to 100% considerable heterogeneity. Funnel plots of standard error versus effect size were generated as exploratory visual tools to assess potential small-study effects. Results of quantitative comparisons were presented using forest plots.

A study-level summary indicating which included experiments contributed to each quantitative meta-analysis outcome (enamel thickness and mineralization parameters) is provided in Supplementary Table S2.

## Results

We reviewed twelve studies^23^, ^24^, ^25^, ^26^, ^27^, ^28^, ^29^, ^30^, ^31^, ^32^, ^33^, ^34^ that analyzed the effects of NSAIDs, acetaminophen, and various antibiotics alone or with NaF on enamel matrix deposition and biomineralization during enamel biogenesis. Study characteristics are summarized in Table 1.

Of the included studies, only *in vivo* animal experiments contributed to the quantitative synthesis, while *in vitro* studies informed mechanistic and qualitative interpretation only. Given the methodological heterogeneity across included studies, the results are presented thematically and categorized according to drug class and outcome domain. Specifically, outcomes are grouped under ameloblast morphology, enamel matrix protein and enzyme alterations, enamel mineralization, and enamel thickness. This structured presentation facilitates clearer interpretation across diverse experimental designs and exposure models.

### Effects of antibiotics on ameloblast morphology (Histopathological findings)

The impact of amoxicillin intake on enamel formation during the secretory and early mineralization stages of amelogenesis has been the focus of a number of studies (Table 1). Early investigations, such as those by Gottberg and colleagues (2014),^33^ explored this by examining the dose-dependent prenatal effects of amoxicillin on dental enamel in Wistar rats. They confirmed that higher doses of amoxicillin resulted in more pronounced alterations in the enamel matrix, highlighting the importance of dosage in the extent of these effects.

Subsequently, De Souza et al^30^ also reported specific effects of amoxicillin on the structural integrity of ameloblasts during the secretory and early mineralization phases. They observed vacuolar structures within ameloblasts, indicating the detrimental effect of amoxicillin on protein secretion and transport processes vital for enamel matrix formation. Moreover, they noted a significant reduction in enamel thickness in amoxicillin-treated rats compared to controls and speculated that the drug`s adverse effects are more pronounced during the initial stages of amelogenesis. These observations were later confirmed by Mihalaş and colleagues^31^ through histopathological studies of the affected enamel matrix. They reported noticeable changes in the mature ameloblasts, including increased cytoplasmic vacuolation, elongated nuclei, and less condensed and amorphous enamel matrix of the teeth of the ‘drugged’ rats. Additionally, unlike the control group, they observed transitional ameloblasts underlying cyst-like lesions of varied sizes and detachment of ameloblasts from the enamel matrix.

Building on the foregoing studies, Kameli et al^29^ postulated that amoxicillin disrupts amelogenesis by interfering with the functionality and survival of ameloblasts. They surmised that such disruption likely stems from the influence of amoxicillin on the molecular pathways that regulate the secretion and maturation of the enamel matrix, as well as interference with protein synthesis mechanisms essential for developing a healthy enamel structure. In addition, they also noted disturbances in enamel and dentin thickness, indicative of disruptions in the early and later stages of enamel maturation.

Later, in 2020, Gao and colleagues^28^ also attempted to investigate the effects of amoxicillin on enamel development, focusing specifically on the secretory and maturation stages. They observed reduced expression of tight junction proteins such as CLDN1, CLDN4, and OCLN in mature ameloblasts, subsequently influencing cellular tight junctions during enamel maturation. This reduction probably impacted the paracellular permeability and the microenvironment essential for enamel mineralization. Moreover, they noted a decreased KLK4 enzyme expression responsible for matrix protein degradation, which, together with alterations in cellular structure, could have contributed to the decreased calcium-to-phosphorus (Ca/P) ratio observed in the enamel of mandibular incisors and molars reported in their study.

Most recently, Sabah and Al Ghaban,^25^ also documented structural alterations in the ameloblasts of rats exposed to amoxicillin during the early stages of amelogenesis, including reduced height, of ameloblasts altered cellular polarization, and the formation of a multicellular layer with deficient enamel matrix production. These perturbations that inhibit the natural differentiation and functionality of ameloblasts are essential for normal enamel development.

Histological examinations provided visual evidence of these disruptions, such as hypomineralized zones near the dentin-enamel junction, vacuolization in the ameloblastic and odontoblastic layers, and decreased enamel and the underlying dentin thickness.

Collectively, these studies elucidate the various mechanisms by which amoxicillin can adversely affect amelogenesis. They highlight a critical window period during the secretory phase when these effects are most pronounced and emphasize the importance of antibiotic dosage and exposure period in determining the severity of these quantitative and qualitative enamel defects.

#### The effect of amoxicillin and amoxicillin+clavulanic acid. (AMC), either alone or in combination with fluoride on amelogenesis

Recent research has investigated the effects of amoxicillin, both alone and in combination with other agents such as clavulanic acid (AMC), on the functionality of ameloblasts during tooth development. Sahlberg et al,^32^ tested varying concentrations of amoxicillin either alone or in combination with sodium clavulanate and NaF and noted that amoxicillin dose-dependently either alone or in combination with NaF impeded the transition of ameloblasts from the secretory to the maturation stage. They observed, on histopathological examination, the presence of multiple vacuoles in the developing ameloblasts on exposure to higher concentrations of amoxicillin, alone or in combination with NaF, indicative of a severe disruption in ameloblast organization and enamel structure. Other interesting observations included increased enamel porosity in the developing first molars at the exposure dosages between 2 and 3.6 mg/mL amoxicillin. Conversely, enamel formation in the second molars was significantly retarded even at lower dosages (1 or 2 mg/mL) of amoxicillin.

Later, Feltrin and team^27^ repeated a similar study in rats and confirmed the adverse effect of these drugs on amelogenesis during the critical developmental stages of tooth development. Taken together foregoing indicates that the combination of the antibiotic and fluoride exposure potentiates their adverse effects on enamel development.

#### Ampicillin and Gentamicin affect the secretory stage of enamel biogenesis

In general, both ampicillin and gentamicin are very effective against a wide range of Gram-positive and Gram-negative bacteria, and they are widely prescribed during the neonatal period and up to age five for management of clinical sepsis.^35^ In 2022, Schmalfuss et al^26^ explored the impact of ampicillin and gentamicin on tooth development in neonatal mice, focusing on their effects on two critical stages, the early secretory and subsequent stages of amelogenesis. They observed notable changes in enamel structure when antibiotics were administered during the secretory stage, adversely affecting enamel volume and highlighting medication-induced disruption of amelogenesis. Regarding enamel quality, the study reported lower mineral density in most segments of the treated incisors, suggesting that the primary mechanism involves the disturbance of enamel mineralization.

#### Macrolides – effects on ameloblasts from the transition to the enamel maturation stages

Abe and colleagues^34^ examined the effects of a new macrolide group drug (belonging to the same family as erythromycin) on enamel and dentine development in rats. They noted significant disruption of enamel development and maturation processes of the developing teeth, reporting that the drug primarily interferes with mechanisms affecting cellular protein synthesis. The disruption was particularly pronounced during the critical transitional stage, where ameloblasts undergo morphological changes from taller, cylindrical shapes to shorter, cuboidal forms, facilitating the mineralization of the newly deposited enamel matrix. They surmised that the observed necrosis of ameloblasts would impair the resorption of the enamel matrix machinery needed at these critical stages of tooth development.^35^

### Effects of antibiotics on enamel thickness – quantitative data

**Fig. 2:**
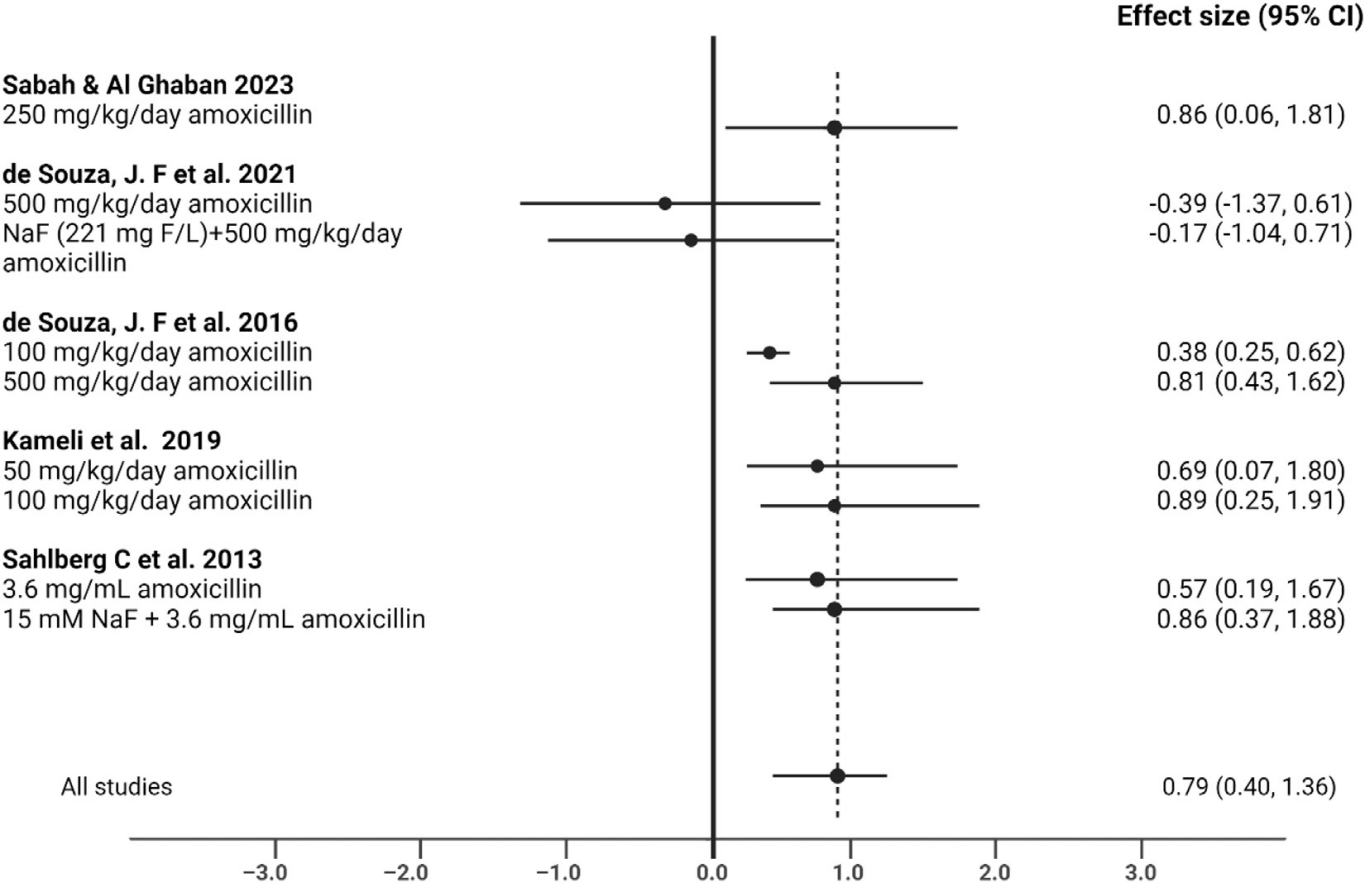
Forest plot of effect sizes for the impact of Amoxicillin/AMX+clav./NaF on developing enamel thickness in various studies.

When evaluating the effect of antibiotics on enamel matrix thickness across all reviewed studies, seven studies (over 55%) demonstrated effect sizes indicating enamel thinning, with Cohen’s d values ranging from −0.39 to 0.87. A forest plot illustrating the individual effect sizes, random-effects mean Cohen’s d, and associated 95% confidence intervals suggests a consistent direction of effect toward reduced enamel thickness, particularly in relation to different concentrations of amoxicillin (Figure 2). Moreover, higher concentrations of amoxicillin, especially in combination with NaF, were associated with larger effect sizes, indicating greater reductions in enamel thickness compared with controls (Figure 2).

Overall, the impact of antibiotic administration was heterogeneous, with substantial inconsistency across studies (*I²* = 75.95%, 95% CI: 67.02-82.37). The average effect size across all studies was 0.79 (95% CI: 0.40-1.36), indicating a moderate-to-large pooled effect estimate, interpreted cautiously in the presence of substantial heterogeneity.

### Effects of antibiotics on enamel biomineralization – qualitative effects

Some investigators have compared the effects of antibiotics and non-steroidal anti-inflammatory drugs (NSAIDs) on enamel maturation.^24^ In a landmark study, Serna Muñoz et al^24^ demonstrated that antibiotic administration did not lead to a significant change in calcium and phosphorus content compared to controls. However, a commonly prescribed non-NSAID, acetaminophen, significantly decreased mineral levels of the maturing enamel despite no notable differences in the calcium and phosphorus ratios. Furthermore, acetaminophen significantly reduced the amount of immunoreactive COX2 of the enamel organ of mouse incisors during the maturation stage.

Similarly, Gonçalves et al^23^ explored the effects of NSAIDs, celecoxib, and indomethacin on developing enamel during its secretion and maturation phases. They reported that these drugs impair the synthesis of critical proteins and enzymes essential for enamel formation, such as MMP-20 and the transcription factor Runx2. This reduction in enamel calcium and phosphorus ratios compromised the microhardness and mineral density of the developing tooth.

**Fig. 3:**
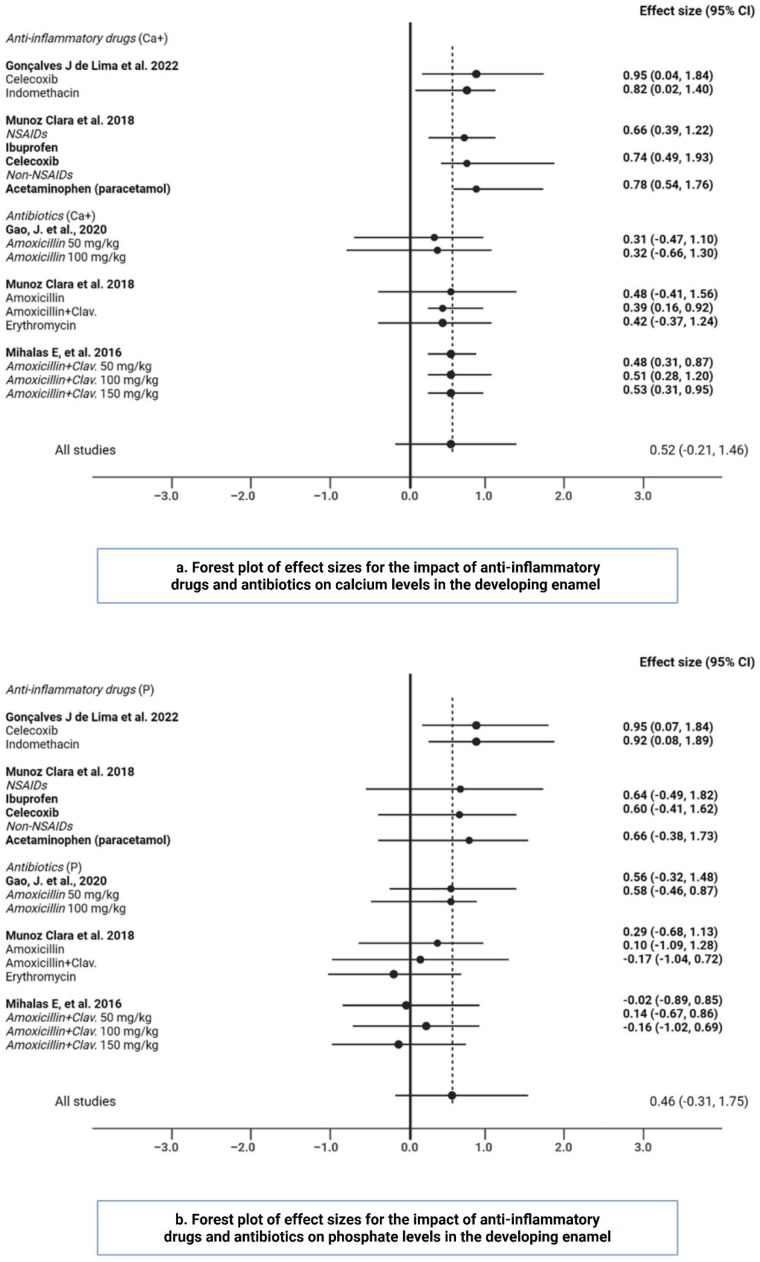
a and b. Forest plot of effect sizes for the impact of anti-inflammatory drugs and antibiotics on Ca+ and P levels in the developing enamel.

Our analysis of the forest plots illustrates the impact of various antibiotics and anti-inflammatory medications on calcium and phosphate enamel content. The overall effect size for Ca^2+^ across all studies was 0.52 (95% CI: −0.21 to 1.46), indicating a non-significant impact (Figure 3a). However, specific drugs, such as celecoxib and indomethacin, showed large effect sizes on the Ca^2+^ content of the developing enamel. Commonly prescribed medications to young children, such as ibuprofen and acetaminophen, demonstrated mixed results related to both Ca^2+^ and P mineral deposition in enamel (Fig. 3, Fig. 3). These data suggest that while certain anti-inflammatory drugs and antibiotics may influence enamel content, the overall effect remains inconclusive.

**Fig. 4:**
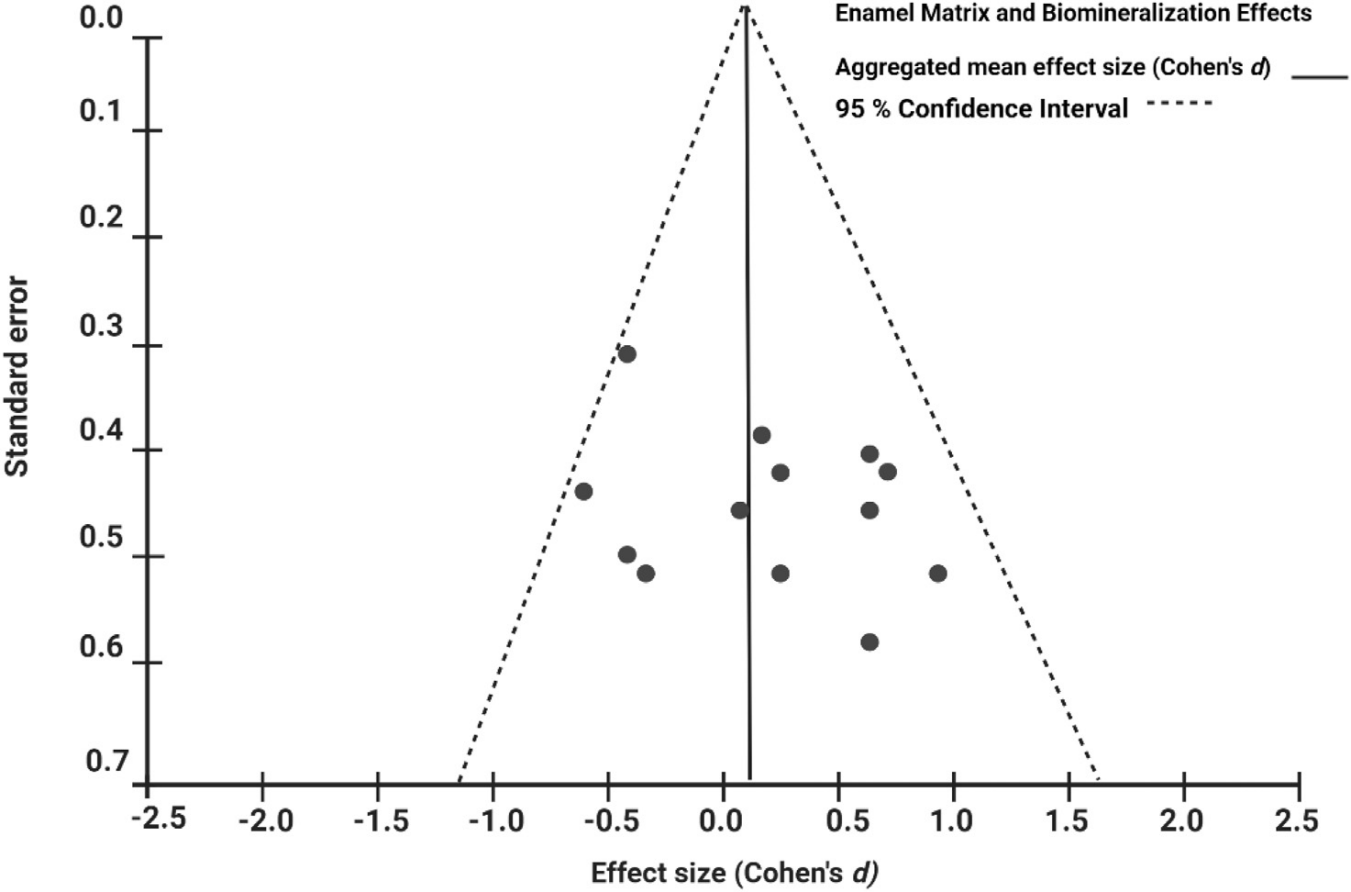
**Funnel plot of effect sizes for enamel thickness and biomineralization outcomes.** Due to fewer than 10 studies per outcome, the funnel plot is presented as an exploratory visual assessment only and not as a definitive test of publication bias.

Overall, the sample size of our review was satisfactory, as the number of test subjects ranged from 5 to 265 per test group across studies. A funnel plot was generated as an exploratory visual assessment of small-study effects for enamel thickness and mineralization outcomes (Figure 4). The number of studies contributing to each outcome ranged from 5 to 7; therefore, formal statistical tests for publication bias (eg, Egger’s or Begg’s tests) were not performed due to insufficient power, and the funnel plot was interpreted cautiously.

## Discussion

This review examines in depth the impact of antibiotics and anti-inflammatory medications prescribed during antenatal and postnatal periods on dental enamel development, particularly in children under five. To our knowledge, this is the first comprehensive review focusing on this specific area. Our review illustrates how commonly prescribed antibiotics can influence enamel biogenesis, resulting in quantitative and qualitative dental anomalies that may persist long-term in affected individuals.

Although a random-effects model was applied, substantial heterogeneity (*I²* = 76%) was observed in the quantitative synthesis. Formal subgroup or meta-regression analyses based on drug type, dose, species, age at exposure, fluoride co-exposure, route of administration, or study quality were not conducted due to the limited number of studies per subgroup. Consequently, pooled effect estimates should be interpreted as descriptive summaries of effect direction and relative magnitude rather than definitive causal effects.

The discussion below is organized by outcome domain and drug class to facilitate focused interpretation across heterogeneous experimental methodologies.

### Effects of antibiotics on ameloblasts and quantitative enamel defects

The studies reviewed consistently demonstrate that several antibiotics, including amoxicillin, ampicillin, gentamicin, tetracycline, and the macrolides, detrimentally affect ameloblasts and their capacity for enamel formation, resulting in reduced enamel thickness.

During the secretory stage, ameloblasts synthesize and secrete multiple structural enamel matrix proteins, which contribute significantly to the overall thickness and volume of enamel.^8^ Histological findings from several reviewed studies elucidate that antibiotics can significantly disrupt the structure and function of ameloblasts. These disruptions manifest as karyopycnotic changes,^34^ vacuoles covering the enamel surface between the poorly organized ameloblasts,^25^^,^^32^ and alterations in odontoblastic and ameloblastic thickness.^25^^,^^26^^,^^29^^,^^30^ Such changes suggest a correlation between medication exposure and disrupted enamel matrix formation.

### Effects of antibiotics on ameloblasts and qualitative enamel defects

Several reviewed studies indicated that antibiotic administration during dental development led to qualitative enamel defects.^28^^,^^31^^,^^33^ Several explanations could be offered for these observed enamel aberrations. Calcium (Ca^2+^), which is the most abundant ion in teeth, is crucial for ameloblast functionality during the secretory stage. Most Ca^2+^ in the enamel fluid during the secretory stage is bound to 11- to 13-kDa amelogenin-derived proteins.^36^ Studies suggest that binding Ca^2+^ to amelogenins, a group of hydrophobic phosphoproteins, provides a route for the diffusion of Ca^2+^ into the deeper layers of enamel.^36^^,^^37^ Thus, initial precursors of enamel crystals form during the early secretory stage in a protein-rich extracellular environment maintained at near-neutral pH conditions. These crystals extend through the dentin-enamel junction to the ameloblast membrane and throughout the enamel.^36^ Hence, it is reasonable to infer that any adverse effects of antibiotics on the secreted amelogenins and related proteins may indirectly impact the biomineralization process.

**Fig. 5:**
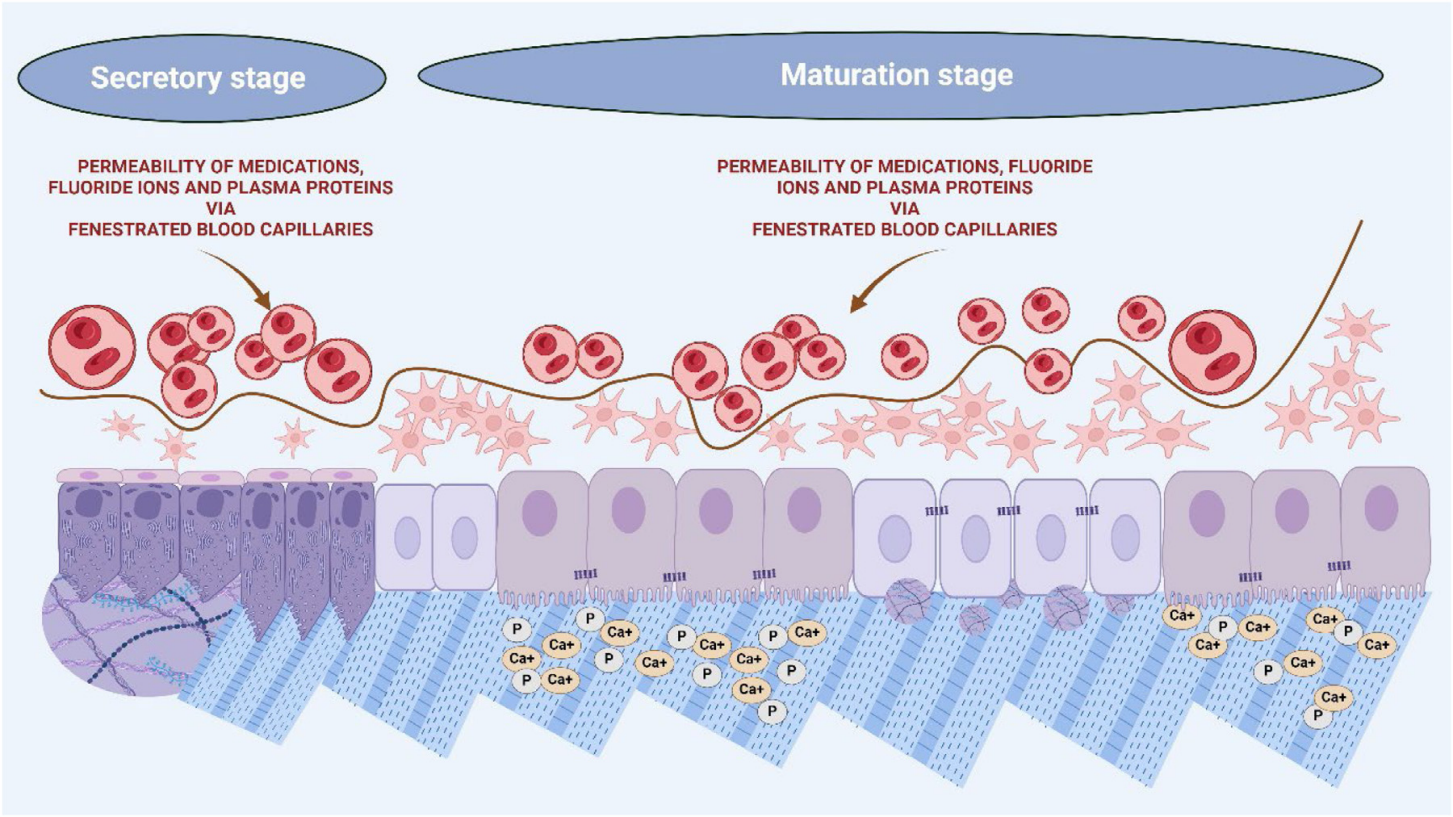
**Increased vascular access during enamel maturation enables drug percolation and potential disruption of mineralization.** Schematic representation of the disappearance of the stellate reticulum and the approach of fenestrated capillaries toward the ameloblast layer during the enamel maturation stage. As blood vessels come into proximity with the ameloblasts, the barrier to systemic substances weakens, increasing the likelihood of medication percolation into the enamel matrix. This enhanced vascular access may facilitate the entry of antibiotics or anti-inflammatory drugs, potentially disrupting enamel mineralization.

Nonetheless, it is during the maturation stages, following the secretory phase, that Ca^2+^ mineral uptake increases significantly due to the upregulation of the Ca^2+^ handling machinery.^37^^,^^38^ During this period, approximately 86% of the Ca^2+^ in the enamel is incorporated into the developing enamel tissue.^38^ Therefore, during the maturation stages, these enamel crystals undergo significant expansion in width and thickness, imparting enamel with its characteristic structure, durability, and hardness^38^ (Figure 5).

The mechanism of enamel biomineralization is extremely complex and a unique phenomenon. Histological studies confirmed that adjacent secretory stage ameloblasts are highly polarized cells tightly opposed and connected by intercellular junctional complexes on the lateral membrane at both the proximal/basal and distal/apical poles.^39^^,^^40^ These junctional complexes form a semipermeable barrier, regulating the intercellular movement and diffusion of mineral ions from the circulation to the enamel matrix.^40^ Mihalaş et al,^31^ noted that commonly prescribed amoxicillin-clavulanate disrupts the expression of such tight junction proteins in mature ameloblasts, disturbing enamel maturation. Changes in the junctional complexes of ameloblasts may also affect paracellular permeability and the microenvironment, likely bearing upon enamel mineralization.^40^

### Effects of antibiotics and anti-inflammatory drugs on cyclooxygenases and enamel quality

The constitutive and inducible enzymes, cyclooxygenases COX-1 and COX-2, respectively, catalyze the formation of prostaglandins from arachidonic acid, mediating several physiological processes, including inflammation.^41^ The induction of COX-2 rapidly augments intracellular levels of nitric oxide, cytokines, and Ca2+ influx.^42^ It could be hypothesized that enamel mineralization requires inflammatory mediators due to the high ion influx needed by ameloblasts during crystal formation,^36^ and indeed, it is known that COX-2 appears to play a physiological role in enamel development.^43^

Nonsteroidal anti-inflammatory drugs exert their anti-inflammatory effects through COX inhibition, which can be either selective or non-selective.^44^ Nonselective inhibitors, such as indomethacin, inhibit COX-1 and COX-2, whereas selective inhibitors like celecoxib specifically inhibit COX-2. On the contrary, ibuprofen, a common NSAID, shows lower potency in COX-2 inhibition.^44^ While acetaminophen, a non-NSAID, also acts as a preferential COX-2 inhibitor.^45^^,^^46^ The findings of Serna Muñoz et al^24^ reported here demonstrate medium to high level suppression of COX2 by ibuprofen and acetaminophen in rats, affecting enamel mineralization.

In the reviewed literature,^23^^,^^24^ we noted that both NSAIDs and non-NSAIDs appear to have a relationship with COX-2 inhibition, leading to a quantitative reduction in minerals of developing tooth enamel, specifically calcium and phosphorus. It is well established that inhibition of COX-2 activity can decrease prostacyclin, which normally stimulates vasodilation and inhibits platelet aggregation.^47^ Thus, it can be hypothesized that a decrease in prostacyclin may reduce blood flow to dental organs, potentially diminishing the delivery of nutrients and the influx of ions and thereby affecting the developing enamel quality. It is noteworthy that drugs such as indomethacin and ibuprofen are commonly prescribed medications to young children.

The relationship between COX-2 inhibition and enamel mineral content highlights the importance of these elements in the mineralization process, suggesting that any disruption in their incorporation is likely to adversely affect amelogenesis.

Our review noted that over a month-long administration of either penicillin or macrolide medications was associated with suppression of the COX2 activity during the enamel maturation stage. In addition to their antibacterial properties, macrolides are known to exhibit immune-modulating and anti-inflammatory effects, possibly via COX2 perturbations.^48^ However, further confirmatory studies are required to understand the long-term effects of penicillin and macrolides and their effects on COX2 enzyme pathways.

The foregoing is particularly pertinent for children on long-term antibiotic prophylactic regimens, such as those with a history of rheumatic fever, congenital heart disease, or conditions predisposing them to recurrent infections. The duration, frequency, and dosage of such treatments must be carefully considered, especially during critical periods of tooth development. Additionally, the suppression of COX2 enzyme activity reported in the reviewed studies confirms the biomineralization effects of these medications on developing enamel, leading to qualitative and or quantitative enamel defects.

#### Fluoride and enamel mineral content in DED

Studies have shown that elemental fluoride exerts a dose-dependent effect on ameloblasts, directly affecting the ameloblasts per se, and matrix maturation.^49^, ^50^, ^51^

As enamel matures, amelogenins, the primary matrix protein, is hydrolyzed by various enamel proteinases, including matrix metalloproteinase-20 (MMP-20 or enamelysin) and serine proteinase.^52^, ^53^, ^54^ Zhang and colleagues (2006) noted in their *in vitro* study that fluoride exposure can modulate the expression of MMP-20 by ameloblasts. This alteration may disrupt the balance between MMP-20 and its substrate, potentially leading to the retention of amelogenins and contributing to the formation of fluorosed enamel.^55^ In agreement with these findings, several reviewed studies^27^^,^^32^ indicate that high fluoride levels, when assessed alongside antibiotics during amelogenesis, lead to a reduction in MMP20 expression,^32^ and modify the enamel morphology and packing of enamel crystallites, ultimately resulting in decreased enamel mineral content.

Assessment of publication bias was limited by the small number of studies per outcome, rendering funnel plots subjective and underpowered for formal statistical testing; consequently, any inference regarding small-study effects should be interpreted with caution.

#### Review limitations

This review is subject to several important limitations. First, substantial methodological heterogeneity was present across the included studies, including variation in animal species, dosing regimens, exposure timing, route of administration, co-exposure to fluoride, and outcome assessment methods. These differences contributed to high between-study heterogeneity and limits the certainty of pooled effect estimates.

Second, all quantitative syntheses were derived exclusively from animal models. Differences in enamel development timelines, gene expression patterns, and mineralization dynamics between animals and humans restrict direct clinical translation. In particular, enamel formation in rodents occurs over a substantially shorter timeframe than in humans, in whom enamel development begins in utero and continues through early childhood. This discrepancy may result in differing exposure windows and biological responses to pharmacological interventions.

Third, the controlled environments of laboratory animal studies do not capture the complex range of environmental, nutritional, systemic, and genetic factors that influence enamel development and susceptibility to dental disease in human populations. Additional uncertainty may also arise from small sample sizes in some studies, digitization of graphical data for quantitative extraction, and a moderate-to-high risk of bias identified in selected methodological domains.

Despite these limitations, the use of animal models remains justified for investigating enamel development, as experimental manipulation of drug exposure during tooth formation would be ethically and practically infeasible in humans. Moreover, mammalian teeth share fundamental structural and developmental features, including an enamel crown formed by epithelial cells, underlying dentin produced by mesenchymal cells, and a vascularized and innervated pulp. Although humans are diphyodonts and rodents are monophyodonts, the continuously growing incisors of rats and mice provide a valuable model for examining all stages of amelogenesis across the lifespan.

Accordingly, the pooled effect sizes reported in this review should be interpreted as exploratory indicators of effect direction and relative magnitude rather than definitive estimates. These limitations highlight the need for cautious interpretation and underscore the importance of future well-designed, standardized experimental and human observational studies to validate and extend these findings.

## Conclusion

This review demonstrates that antibiotics and anti-inflammatory medications can influence enamel development in animal models, leading to both qualitative and quantitative enamel alterations through multiple biological mechanisms.

However, these findings are derived exclusively from experimental animal studies, and direct translation to human clinical prescribing should be approached with caution. While the evidence suggests potential biological vulnerability of developing enamel to pharmacological exposure during critical stages of tooth formation, the results should be considered hypothesis-generating rather than prescriptive for clinical decision-making.

From a clinical and public health perspective, these findings underscore the importance of judicious use of antibiotics and anti-inflammatory medications during early childhood, without implying direct causality or clinical thresholds. Long-term or frequent exposure to broad-spectrum agents warrants further investigation rather than immediate changes to prescribing practice.

Future research should prioritize well-designed human observational studies and mechanistic investigations to validate these experimental findings, clarify dose- and timing-dependent effects, and identify strategies to mitigate potential impacts on enamel development. Improved understanding of the interactions between pharmacological exposures and dental development may ultimately inform safer therapeutic approaches and preventive strategies.

## Author contribution

Nadia Zamri, Hien Ngo, and Kausar S Fakhruddin: involved in study conception, data collection and analysis, drafting and revising the manuscript. Lakshman Samaranayake, Elizebeth Court, and Benette Amaechi contributed to data analysis and editing the manuscript. Saniya Khani assisted with software use and technical writing. Kausar S Fakhruddin and Nadia Zamri were involved in the revision of the manuscript.

## Declaration of generative AI and AI-assisted technologies in the writing process

During the preparation of this work, the authors used Grammarly to assist with grammar correction and sentence structure refinement. The authors subsequently reviewed and edited the content in full and take complete responsibility for the accuracy and integrity of the final manuscript.

## Conflict of interest

The author is an Editorial Board Member/Editor-in-Chief/Associate Editor/Guest Editor for this journal and was not involved in the editorial review or the decision to publish this article.

## Appendix Supplementary materials

Image, application 1

Image, application 2

## Footnotes

Supplementary material associated with this article can be found in the online version at doi:10.1016/j.identj.2026.109478.
