# Supplementary material for: Effects of Antibiotics and Anti-Inflammatory Drugs on Enamel Development: A Systematic Review with Quantitative Synthesis
Source: Int Dent J. 2026 Mar 12;76(3):109478. doi: 10.1016/j.identj.2026.109478 (PMC12997327; doi:10.1016/j.identj.2026.109478)
Supplement: Supplementary file 1 [file mmc1.docx]

**Table S1. Search Terms**

| **PubMed** | 1. ("Dentinogenesis" OR "Dentin, Secondary" OR "Dentin, Tertiary") AND  2. ("Tooth Development" OR "Deciduous Tooth" OR "Permanent Tooth" OR "Odontogenesis") AND  3. ("Dental Enamel" OR "Dental Dentin" OR "Ameloblasts" OR "Odontoblasts") AND  4. ("Bud Stage" OR "Cap Stage" OR "Bell Stage" OR "Morphogenesis") AND  5. ("Amelogenesis" OR "Enamel Matrix" OR "Amelogenin" OR "Enamelin" OR "Tuftelin" OR "Ameloblastin") AND  6. ("Hydroxyapatites" OR "Calcium Phosphates" OR "Biomineralization") AND  7. ("Amoxicillin" OR "Amoxicillin-Potassium Clavulanate Combination" OR "Ampicillin" OR "Gentamicins" OR "Macrolides" OR "Erythromycin" OR "Tetracycline" OR "Azithromycin") AND  8. ("Non-Steroidal Anti-Inflammatory Agents" OR "Acetaminophen" OR "Ibuprofen" OR "Celecoxib") AND  9. ("Dental Enamel Hypoplasia" OR "Enamel Microstructure" OR "Enamel Thickness" OR "Enamel Mineralization") AND |
| --- | --- |
| **Web of Science** | TS=("Dentinogenesis" OR "Dentin, Secondary" OR "Dentin, Tertiary") AND  TS=("Tooth Development" OR "Deciduous Tooth" OR "Permanent Tooth" OR "Odontogenesis") AND  TS=("Dental Enamel" OR "Dental Dentin" OR "Ameloblasts" OR "Odontoblasts") AND  TS=("Bud Stage" OR "Cap Stage" OR "Bell Stage" OR "Morphogenesis") AND  TS=("Amelogenesis" OR "Enamel Matrix" OR "Amelogenin" OR "Enamelin" OR "Tuftelin" OR "Ameloblastin") AND  TS=("Hydroxyapatites" OR "Calcium Phosphates" OR "Biomineralization") AND  TS=("Amoxicillin" OR "Amoxicillin-Potassium Clavulanate Combination" OR "Ampicillin" OR "Gentamicins" OR "Macrolides" OR "Erythromycin" OR "Tetracycline" OR "Azithromycin") AND  TS=("Non-Steroidal Anti-Inflammatory Agents" OR "Acetaminophen" OR "Ibuprofen" OR "Celecoxib") AND  TS=("Dental Enamel Hypoplasia" OR "Enamel Microstructure" OR "Enamel Thickness" OR "Enamel Mineralization") AND |
| **EMBASE** | ('dentinogenesis' OR 'dentin, secondary' OR 'dentin, tertiary') AND  ('tooth development' OR 'deciduous tooth' OR 'permanent tooth' OR 'odontogenesis') AND  ('dental enamel' OR 'dental dentin' OR 'ameloblasts' OR 'odontoblasts') AND  ('bud stage' OR 'cap stage' OR 'bell stage' OR 'morphogenesis') AND  ('amelogenesis' OR 'enamel matrix' OR 'amelogenin' OR 'enamelin' OR 'tuftelin' OR 'ameloblastin') AND  ('hydroxyapatites' OR 'calcium phosphates' OR 'biomineralization') AND  ('amoxicillin' OR 'amoxicillin-potassium clavulanate combination' OR 'ampicillin' OR 'gentamicins' OR 'macrolides' OR 'erythromycin' OR 'tetracycline' OR 'azithromycin') AND  ('non-steroidal anti-inflammatory agents' OR 'acetaminophen' OR 'ibuprofen' OR 'celecoxib') AND  ('dental enamel hypoplasia' OR 'enamel microstructure' OR 'enamel thickness' OR 'enamel mineralization') AND  ('animal experimentation' OR 'histological techniques') |
| **CINAHL** | MH ("Dentinogenesis" OR "Dentin, Secondary" OR "Dentin, Tertiary") AND  MH ("Tooth Development" OR "Deciduous Tooth" OR "Permanent Tooth" OR "Odontogenesis") AND  MH ("Dental Enamel" OR "Dental Dentin" OR "Ameloblasts" OR "Odontoblasts") AND  MH ("Bud Stage" OR "Cap Stage" OR "Bell Stage" OR "Morphogenesis") AND  MH ("Amelogenesis" OR "Enamel Matrix" OR "Amelogenin" OR "Enamelin" OR "Tuftelin" OR "Ameloblastin") AND  MH ("Hydroxyapatites" OR "Calcium Phosphates" OR "Biomineralization") AND  MH ("Amoxicillin" OR "Amoxicillin-Potassium Clavulanate Combination" OR "Ampicillin" OR "Gentamicins" OR "Macrolides" OR "Erythromycin" OR "Tetracycline" OR "Azithromycin") AND  MH ("Non-Steroidal Anti-Inflammatory Agents" OR "Acetaminophen" OR "Ibuprofen" OR "Celecoxib") AND  MH ("Dental Enamel Hypoplasia" OR "Enamel Microstructure" OR "Enamel Thickness" OR "Enamel Mineralization") AND |
| **SCOPUS Database** | TITLE-ABS-KEY("Dentinogenesis" OR "Dentin, Secondary" OR "Dentin, Tertiary") AND  TITLE-ABS-KEY("Tooth Development" OR "Deciduous Tooth" OR "Permanent Tooth" OR "Odontogenesis") AND  TITLE-ABS-KEY("Dental Enamel" OR "Dental Dentin" OR "Ameloblasts" OR "Odontoblasts") AND  TITLE-ABS-KEY("Bud Stage" OR "Cap Stage" OR "Bell Stage" OR "Morphogenesis") AND  TITLE-ABS-KEY("Amelogenesis" OR "Enamel Matrix" OR "Amelogenin" OR "Enamelin" OR "Tuftelin" OR "Ameloblastin") AND  TITLE-ABS-KEY("Hydroxyapatites" OR "Calcium Phosphates" OR "Biomineralization") AND  TITLE-ABS-KEY("Amoxicillin" OR "Amoxicillin-Potassium Clavulanate Combination" OR "Ampicillin" OR "Gentamicins" OR "Macrolides" OR "Erythromycin" OR "Tetracycline" OR "Azithromycin") AND  TITLE-ABS-KEY("Non-Steroidal Anti-Inflammatory Agents" OR "Acetaminophen" OR "Ibuprofen" OR "Celecoxib") AND  TITLE-ABS-KEY("Dental Enamel Hypoplasia" OR "Enamel Microstructure" OR "Enamel Thickness" OR "Enamel Mineralization") AND |
