# Supplementary material for: Effects of Antibiotics and Anti-Inflammatory Drugs on Enamel Development: A Systematic Review with Quantitative Synthesis
Source: Int Dent J. 2026 Mar 12;76(3):109478. doi: 10.1016/j.identj.2026.109478 (PMC12997327; doi:10.1016/j.identj.2026.109478)
Supplement: Supplementary file 2 [file mmc2.docx]

**TABLE S2.** Studies contributing to qualitative synthesis and quantitative meta-analysis outcomes

| **Study (Year)** | **Model** | **Outcome(s) Assessed** | **Included in Meta-analysis** |
| --- | --- | --- | --- |
| Sabah & Al Ghaban, 2023 | *In vivo* (rat) | Enamel thickness, morphology | Yes (thickness) |
| Gonçalves et al., 2022 | *In vivo* (mouse) | Ca, P, microhardness | Yes (Ca, P) |
| Schmalfuss et al., 2022 | *In vivo* (mouse) | Enamel volume/thickness | Yes (thickness) |
| Feltrin de Souza et al., 2021 | *In vivo* (rat) | Enamel thickness | Yes (thickness) |
| Gao et al., 2020 | *In vivo* (mouse) | Ca/P ratio, morphology | Yes (Ca, P) |
| Kameli et al., 2019 | *In vivo* (rat) | Enamel thickness | Yes (thickness) |
| Munoz Clara et al., 2018 | *In vivo* (mouse) | Ca, P | Yes (Ca, P) |
| de Souza et al., 2016 | *In vivo* (rat) | Enamel thickness | Yes (thickness) |
| Mihalas et al., 2016 | *In vivo* (mouse) | Ca, P, morphology | Yes (Ca, P) |
| Gottberg et al., 2014 | *In vivo* (rat) | Ameloblast morphology | No (qualitative only) |
| Sahlberg et al., 2013 | *In vitro* (tooth germ culture) | Enamel thickness, morphology | No (qualitative only) |
| Abe et al., 2003 | *In vivo* (rat) | Ameloblast morphology | No (qualitative only) |
